# Impact of a Teaching Kitchen Curriculum for Health Professional Trainees in Nutrition Knowledge, Confidence, and Skills to Advance Obesity Prevention and Management in Clinical Practice

**DOI:** 10.3390/nu15194240

**Published:** 2023-09-30

**Authors:** Christine K. Thang, Alma D. Guerrero, Cambria L. Garell, Janet K. Leader, Erica Lee, Kevin Ziehl, Catherine L. Carpenter, Shanika Boyce, Wendelin Slusser

**Affiliations:** 1 Department of Pediatrics, University of California Los Angeles, Los Angeles, CA 90095, USA; aguerrero@mednet.ucla.edu (A.D.G.); cgarell@mednet.ucla.edu (C.L.G.); wslusser@conet.ucla.edu (W.S.); 2 Department of Community Health Sciences, UCLA Fielding School of Public Health, Los Angeles, CA 90095, USA; jleader30@hotmail.com; 3 Department of Biostatistics, UCLA School of Public Health, Los Angeles, CA 90095, USA; kevinziehl@ucla.edu; 4 UCLA Center for Human Nutrition, Schools of Medicine, Nursing and Public Health, Los Angeles, CA 90095, USA; ccarpenter@mednet.ucla.edu; 5 Department of Pediatrics, Charles R. Drew University of Medicine and Science, Los Angeles, CA 90095, USA; shanikaboyce@cdrewu.edu; 6 Semel Healthy Campus Initiative Center, UCLA, Los Angeles, CA 90095, USA

**Keywords:** teaching kitchen, nutrition curriculum, health professionals training, medical education, resident physicians, nursing students, medical students, dental students

## Abstract

Nutrition knowledge, confidence, and skills are thought to be important elements in the role of healthcare professionals in obesity prevention and management. The Upstream Obesity Solutions curriculum goes upstream with a multidisciplinary approach to supplement nutrition education among health professional trainees. Educational strategies of didactics, teaching kitchens, and service-based learning were employed for medical, dental, and nursing students and resident physicians. Pre/post participation surveys assessed knowledge, attitude, and practices; lifestyle habits; and culinary skills among 75 trainees in this cross-sectional descriptive study. There was variability in statistically significant improvement in knowledge, attitudes, and practices about obesity management and nutrition education, lifestyle habits, and culinary skills among learner groups.

## 1. Introduction

The prevalence of Americans with elevated body mass indexes in the United States (US) was 18.5% among youth and 39.8% among adults, as noted by the US Centers for Disease Control and Prevention National Center for Health Statistics in 2017 [1,2]. There is a clear need to build capacity at the primary and tertiary care levels to address obesity in the US, and this has been a priority area for the US government. Counseling on healthy eating and physical activity behaviors is an evidence-based recommendation to prevent obesity by maintaining energy balance and to manage obesity by decreasing energy intake and increasing energy expenditure [3,4]. Yet, most healthcare professionals (HCP) report receiving insufficient training in effective nutrition and lifestyle counseling and feel ill-equipped to manage obesity in the primary care setting [5,6,7,8,9]. Thus, many HCP may be ineffectively prescribing nutrition recommendations for obesity prevention and management [10].

Given the role different HCP play in nutrition counseling, training programs must keep pace with nutrition education. Nutrition education is complex and diverse including the factors and contexts that influence the individual, environment, society, and culture. The social-ecological model illustrated in the USDA Dietary Guidelines for Americans attempts to explain how these factors combine to shape dietary behaviors [11]. Thus, training considerations must also acknowledge and address the complexity of nutrition education, attempt to integrate some of the determinants of nutrition, and include cultural humility, food justice, and social determinants.

Furthermore, current trends in graduate education include merging classroom and clinical training for earlier clinical exposure, more hands-on experiential learning, and small group experiences [8,12,13,14]. Acknowledging the need for nutrition curricula to support these new healthcare training environments, we took an upstream approach to HCP training programs and turned to culinary medicine to integrate nutrition education, obesity management, and skill-building for providers to counsel their future patients.

Teaching kitchens are an emerging experiential concept where participants learn evidence-based nutrition information, culinary skills, self-care practices, and other subjects in a hands-on kitchen environment. A study at Tulane University found nutrition education through a teaching kitchen significantly increased medical students’ competencies in patient nutrition counseling [14]. Various institutions have started teaching kitchens to address unmet nutrition education needs through the national Teaching Kitchen Collaborative [15].

Building upon the successes of these other teaching kitchen curricula in the literature, the Upstream Obesity Solutions (UOS) Teaching Kitchen curriculum was implemented at an academic university center. It was informed by a 2017 pilot involving a small cohort of HCP students, which demonstrated significant improvements in culinary skills and confidence in advising patients about nutritional habits and meal preparation. Given the success of the pilot, we proceeded to disseminate the curriculum to a larger group of HCP trainees. In this report, we describe how we conducted the UOS Teaching Kitchen curriculum with trainees from three disciplines (medicine, dentistry, nursing) and across two levels of training (undergraduate and graduate), and the evaluation outcomes of this program. We delivered the training across these disciplines given the recognized role these health professionals play in nutrition counseling.

The UOS program utilizes the conceptual framework of experiential learning to train the next generation of HCP with the knowledge, skills, and confidence in culinary skills and nutrition counseling. The aim of this study is to investigate the impact of the UOS program on the knowledge acquisition, attitudinal shifts, and practical application of healthcare trainees pertaining to nutrition education, lifestyle habits, and culinary skills.

## 2. Materials and Methods

The UOS team included university faculty and staff members with expertise in medical, nursing, nutrition, public health, culinary, and dental education and representing diverse disciplines including physicians, researchers, chefs, dietitians, and health sciences students. This team collaborated to develop the UOS curriculum. Learning objectives and educational content were based on national guidelines, existing literature, and key informant interviews. We considered learners from different disciplines and training levels to develop an educational product that would be adaptable across specialties and training levels. Lastly, we integrated experiential learning with a train-the-trainer model for an interactive approach centered on the HCP trainees as the targeted learners [16].

### 2.1. Educational Modalities

Given the commonly encountered barrier of time limitations within the different professional schools, we employed multiple modalities to deliver the educational curriculum while ensuring the three core components were similar across all courses. The educational approach included hands-on learning coupled with a train-the-trainer model to equip trainees with the knowledge, attitude, and skills to promote healthy lifestyles for themselves and their patients. The aim was for trainees to ultimately share and apply their gains in knowledge and skills with future patients utilizing the “see one, do one, teach one” methodology common across training.

The three core components across all the courses were the following: the teaching kitchen experience, the service-based learning experience, and the didactic session. The teaching kitchen experience encompassed a series of two-hour live hands-on interactive cooking lesson delivered by a professional chef and registered dietitian. Each course used the same lesson plan that included nutrients, foods, and recipes associated with the prevention of chronic disease and the promotion of healthy meals based on the US 2015–2020 Dietary Guidelines for Americans. Each course also used the same teaching kitchen skills lessons to prepare healthy meals such as knife skills and reading nutrition labels.

The second core component was a service-based learning experience that consisted of newly trained learners delivering their recently acquired culinary and nutrition skills in a cooking demonstration to patients and their families at a federally qualified health center. Lastly, the third component was didactic sessions designed for synchronous and asynchronous learning and accomplished using traditional lectures and self-study modules (e.g., podcasts, PowerPoint presentations, mobile device applications, and interactive online modules). Table 1 outlines the course content and educational modalities employed for each cohort of learners in the UOS program.

The UOS team delivered the multidisciplinary curriculum through four courses. All sessions occurred during the 2019–2020 academic year. The first was the Spring Sizzle, a five-week not-for-credit course offered through the Medical School and Dental School composed of first and second year medical and dental students. Students voluntarily enrolled in the program and completed the self-study modules, the live teaching kitchen, and the service-learning kitchen.

Next, the “Improving Health for You and Your Patients Selective” (Selective) was offered through the Medical School. Medical students from their first and second year of training electively enrolled in the course and received academic credit toward graduation. Over the seven-week elective, they received lectures, self-study modules, the teaching kitchen hands-on sessions, and the service-learning kitchen experience with children and families one afternoon per week.

The “Public Health and Ambulatory Basics and Beyond” (PHABB) was a two-week elective rotation in the Pediatric Residency Training Program. Pediatric resident physicians were enrolled during their second year of training. Over the two weeks, the UOS curriculum was integrated so resident participants received didactic lectures, self-study modules, the teaching kitchen lessons and hands-on learning, and the service-learning kitchen experience.

Lastly, the core components of the UOS curriculum were integrated into “Health Promotion: Nutrition,” a required course in the Nursing School. Over the 10-week course, nursing students were required to attend teaching kitchen sessions. The nursing students then delivered their respective teaching kitchen lesson to their classmates on the last day of class as their service-learning kitchen experience.

### 2.2. Participants

Study participants were HCP trainees from the aforementioned undergraduate and graduate healthcare training programs. The recruitment varied across the different programs as participants enrolled through required or elective courses. All sessions occurred during the 2019–2020 academic year. Participants included in the data analyses were learners who completed their pre- and post-course surveys. We combined the Spring Sizzle and the Selective groups for certain data analyses because of the small number of participants and the similar core components of the curricula across the groups. Informed consent was obtained from all participants involved in the study. All procedures involving research study participants were approved by the University of California Los Angeles Institutional Review Board (UCLA IRB). The UCLA IRB’s Federalwide Assurance (FWA) with Department of Health and Human Services is FWA00004642 (IRB00004474).

### 2.3. Survey Design

Course surveys were created by the investigators using portions of existing surveys published in the literature since no single validated tool was found that met the needs of the study [5,15,17,18,19,20,21,22,23,24,25]. The developed survey instrument measured changes in knowledge, attitudes, and practices (KAP) about obesity management and nutrition education, lifestyle habits, and culinary skills. The KAP survey evaluated knowledge through a series of multiple-choice questions about obesity and nutrition, concepts that were covered in the teaching kitchens, and we tabulated the total number of questions answered correctly. Several of the multiple-choice items were based on a prior assessment evaluating a pediatric obesity curriculum in another residency program [20], self-assessment questions from a pediatric obesity review [21], and a previously published evaluation survey of medical students who participated in a teaching kitchen educational experience [22]. The KAP survey also looked at attitudes and beliefs about the following: “Provider’s Role in Managing Obesity” [5], “Confidence in Obesity Knowledge” [17,18], and “Confidence in Counseling Families about Obesity” [17,18,19] scored on a five-level Likert scale from “Strongly Disagree” to “Strongly Agree.” Questions asked under these themes are included in Supplementary Materials Table S1.

Next, the lifestyle survey was a self-evaluation of the dietary intake, cooking habits, physical activity, sleep, and overall well-being of learners. Questions were adapted from the baseline participant survey of the Teaching Kitchen Collaborative [15]. We measured sleep patterns using a sleep scale to assess the number of hours learners slept and employed the Epworth Sleepiness Scale [23] to assess daytime sleepiness as part of the quality of sleep assessment of the lifestyle survey.

Lastly, the culinary skills survey was adapted from previously published psychosocial scales for evaluating the impact of teaching kitchens and culinary nutrition education programs [24,25]. The culinary skills survey inquired about confidence with cooking skills including knife skills and portion sizes, and confidence with nutrition literacy including reading nutrition labels. Recommendations were based on the published Dietary Guidelines for Americans. The questionnaires are available from the authors upon request.

### 2.4. Evaluation

Pre-course and post-course surveys were administered to the learners enrolled in each course (Table 1). The Spring Sizzle course only received the KAP survey due to its earlier implementation in contrast to the later delivery of the other groups that received all three surveys. On the first day of each course, pre-participation paper surveys were handed out to each participant, and similarly, post-participation surveys on the last day of the course. The study was conducted in accordance with our institutional review board.

### 2.5. Statistical Analysis

Due to variability in implementation for each course because of the different professional schools, we conducted pre/post comparisons within each course only and not across the groups. We compared the paired pre-course and post-course measurements for participants who completed their surveys within each course. For the knowledge assessment portion of the KAP survey, we tabulated the total correct answers both before and after course participation. For the remaining items on the KAP survey, scores were based on participants’ answers to their level of agreement or confidence to each statement on a five-point Likert scale from “Strongly Disagree” to “Strongly Agree.”

Survey data were entered in EXCEL and imported into SAS 9.4^®^ where data cleaning and transformation occurred. We constructed the individual records to reflect measurements taken before the program started and afterward. Matched paired t-tests were used to assess changes in mean survey responses before and after participation. Additionally, Python^®^ was employed to evaluate rank sum mean differences using the Wilcoxon rank test. For categorical and dichotomous data, McNemar Chi-Square was used to assess discordant pairs whose scores had changed from pre-course to post-course compared to concordant pairs whose scores had not changed.

## 3. Results

Study participants were a total of 75 learners who completed both their pre-course and post-course surveys. There were 21 participants from the Spring Sizzle and Selective courses consisting of medical and dental students, 37 nursing undergraduate students from the Health Promotion course, and 17 pediatric resident physicians from the PHABB course. Because of the small number of participants and the similar core components of the curricula, we combined the Spring Sizzle and the Selective groups for certain data analyses. The data presented in the tables report results from the participants with completed surveys.

Table 2 contains results from the KAP survey before and after participation. We observed statistically significant improvement in understanding the “Provider’s Role in Managing Obesity” among pediatric residents (PHABB) (*p* < 0.05), and no improvement among the nursing nor medical/dental students. “Confidence in Knowledge about Obesity” and “Confidence in Counseling about Obesity” significantly improved in all the groups (*p* < 0.05). General knowledge about obesity significantly improved for nursing students and pediatric residents (PHABB) (*p* < 0.05) but not for medical students (Selective).

From the self-reported lifestyle survey, Table 3 presents categorical variables in relation to the frequency of responses before and after participation in the teaching kitchen. The *p*-values in Table 3 represent McNemar-matched paired Chi-Square tests for pre- and post-survey results. Results were not compared across groups because the participants were in different stages of their educational training. The Spring Sizzle course did not have the self-evaluation survey administered. Nursing and medical students (Selective) both resulted in statistically significant improvement in days consuming at least five servings of fruits and vegetables (*p*-value < 0.05). Consumption of whole grains significantly improved in the Selective group but not the others. Pediatric residents (PHABB) showed improved number of hours spent sleeping. “Advising patients about nutrition” and “advising patients about cooking” did not change significantly for any of the groups, nor did minutes per day for moderate exercise.

Table 4 presents distributions of continuous variables from the self-evaluated lifestyle survey according to pre- and post-course. Pediatric residents (PHABB) showed significant improvement in days per week moderately exercising, work-life balance, social well-being, hours slept on the weekend, and reduction in sleepiness. Nursing students showed a significant reduction in hours per week spent sleeping on weekdays. All other changes were not significant. The medical student (Selective) group showed no significant improvement in any of the self-evaluation continuous measures.

From Table 5, all participation groups demonstrated statistically significant improvement in cooking skills and nutrition literacy after their course (*p* < 0.05).

## 4. Discussion

Our program is timely because numerous medical education programs are currently revising their curricula [26]. Addressing obesity is a critical public health issue and guiding effective interventions necessitates a closer look at the training of healthcare providers. Numerous studies emphasize the need for improved nutrition education within this group. The current literature underscores the need for nutrition education for healthcare professional trainees. A national survey conducted by the Nutrition in Medicine Project at the University of North Carolina found only nine medical schools consistently adhering to the recommended 25 hours of nutrition instruction [27]. In a university-based internal medicine training program, 77% of respondents acknowledged the importance of nutrition assessments during primary care visits, yet only 14% felt adequately prepared to offer such guidance [18]. This gap is similarly echoed among practicing healthcare providers, as indicated by a scoping literature review revealing that primary care physicians and nurses tend to provide nutrition counseling primarily when patients’ BMI is on the rise and with often suboptimal quality advice [28].

In this report, we describe the UOS Teaching Kitchen curriculum as an educational pilot aimed at delivering nutritional knowledge and culinary skills to HCP trainees across various disciplines. The different educational strategies UOS utilized seem effective in significantly impacting trainees’ self-efficacy related to counseling; cooking skills; confidence in nutrition knowledge; and nutrition literacy. It has been recognized that patients find motivation in providers sharing their own healthy habits [13]. While the initial aim of the curriculum was to address nutrition education gaps, we leveraged it to equip HCP trainees with practical skills applicable to patient counseling through a train-the-trainer model using experiential learning approaches. Interactions with interdisciplinary faculty members, including physicians, chefs, and dietitians, may have contributed to the observed self-perceived improvements in cooking skills and nutrition literacy among the learners.

The KAP surveys before and after participation revealed variations in responses related to “Provider’s Role in Managing Obesity,” “Confidence in Knowledge about Obesity,” and “Confidence in Counseling about Obesity” among participants. Notably, pediatric residents (PHABB) demonstrated a statistically significant improvement in their understanding of the “Provider’s Role in Managing Obesity.” This outcome may be attributed to their specific training in primary care and their advanced stage of training, aligning their roles more closely with the management of this prevalent primary care health condition.

Furthermore, self-reported lifestyle behaviors of trainees before and after the course exhibited variations in responses. Some differences observed were situational, such as a decline in sleep hours reported by nursing students in the post-survey due to final exam preparation. Similarly, the PHABB group, consisting of pediatric residents, did not show significant changes in fruit, vegetable, and whole grain intake, likely due to their higher baseline consumption of these nutrients. For example, at baseline, 42.9%, 10.5%, and 43.8% of trainees reported consuming 0–1 servings of fruits/vegetables per day among the Nursing, PHABB, and Selective groups, respectively. A similar trend was observed in whole grain intake. The significant change in sleep hours was particularly noticeable in the pediatric resident group, likely due to a reduction in clinical demands during their rotation, resulting in fewer long hours and overnight shifts at the hospital.

We do recognize limitations in the implementation and evaluation of the UOS curriculum. This descriptive cross-sectional report lacked a control group, and the findings cannot be generalized to other higher educational institutions or professional cohorts. Nevertheless, similar findings have been reported in the literature for teaching kitchen curricula, even in the absence of comparison controls [15,22,29]. Furthermore, our study acknowledges the potential variations in the level of involvement in nutrition education and obesity management among different healthcare professionals, which could lead to differences in interest and skill improvement. Our initial assumption was that all participants, being learners in training programs, would share a general interest in and engagement with the program. We conducted comparisons within each group of participants to assess the program’s impact within the specific context of their training programs. However, we did not compare the groups against each other, which could be a subject for further exploration.

Moreover, while we observed short-term improvements in reported lifestyle behaviors, predicting long-term changes is challenging. Trainees’ lifestyle changes may have been influenced by the timing and circumstances surrounding their courses, such as reduced clinical demands or more stressful examination periods. Although all participation groups reported significant improvements in self-assessed cooking skills and nutrition literacy after their completing the educational program, trainees did not report significant changes in their self-reported practices of advising of patients about nutrition or cooking. We suspect that assessing behavioral changes in this context was challenging due to the limited number of clinical encounters that occurred during the course itself. Nevertheless, the significant improvements in self-reported measures of knowledge and confidence are encouraging because it may well translate into actual practice among our learners when they have the opportunity to apply it in a clinical setting.

## 5. Conclusions

In summary, the ability to embed the UOS curriculum into existing courses provides a practical solution in addressing the various scheduling and logistical challenges in organizing and gathering different HCP trainees to learn together in the same setting. Next steps will investigate how the UOS Teaching Kitchen curriculum as an innovative approach to improving nutrition education across different HCP trainees can become a required component of formal training programs.

Regarding our future direction, after successfully delivering the program in 2020, the unforeseen global pandemic prompted us to transition to online learning platforms. In the upcoming phases, we aim to assess and compare the effectiveness of in-person versus online program delivery during the years 2020 to 2022. This analysis will help us refine our approach to meet the evolving needs of our trainees. Additionally, within our institution’s educational landscape, we are actively exploring avenues for broader dissemination of this program. Our team’s presence in faculty roles on the undergraduate campus positions us strategically to expand our program’s reach even further upstream in the educational continuum. Additionally, we continue to advocate for nutrition education becoming a course requirement rather than an elective choice within healthcare professional training programs. The importance of nutrition education has already been brought to the forefront of the curricular redesign efforts within our academic institution. Evaluating the efficiency of remotely conducted training in comparison to live, in-person delivery is a valuable undertaking, particularly as the medical education literature delves into distance learning strategies.

## Figures and Tables

**Table 1:** Course Design.

Course Name	Participants	Educational Modalities	Pre/Post Surveys
Spring Sizzle	Medical Students Dental Students	14 self-study modules Nutrition instruction during Teaching Kitchen 3 Teaching Kitchens Service Learning Experience	Knowledge, Attitude, Practices (KAP)
Improving Health with Your Fork: A Nutrition and Cooking Course for You and Your Patients (Selective)	Medical Students	2 in-class lectures 8 self-study modules Nutrition instruction during Teaching Kitchen 3 Teaching Kitchens Service Learning Experience	KAP Lifestyle Culinary Skills
Health Promotion: Nutrition (Nursing Course)	Nursing Students	9 in-class lectures 1 self-study module Nutrition instruction during Teaching Kitchen 1 Teaching Kitchen	KAP Lifestyle Culinary Skills
Public Health Ambulatory Basics and Beyond (PHABB)	Pediatric Residents	Nutrition instruction integrated into Teaching Kitchen 9 in-class lectures 16 self-study modules 3 Teaching Kitchens Service Learning Experience	KAP Lifestyle Culinary Skills

**Table 2:** Pre-course and post-course survey measuring health profession trainees’ knowledge, attitudes, and practices around nutrition education and obesity management.

Outcome Measure		Pre-Class		Post-Class		*t*-Test	Wilcox
Class Grouping	n	Mean	Std Dev	Mean	Std Dev	*p*-Value ^e^	*p*-Value ^f^
KAP ^a^: Obesity Management (9 questions)							
Sizzle + Selective ^b^	21	41.1	3.2	42.1	2.8	0.17	0.17
Health Promotion in Nursing	37	36.5	4.8	37.2	5.0	0.37	0.37
PHABB	17	39.1	4.7	40.8	2.7	0.03 *	0.03 *
KAP: Confidence in Knowledge (11 questions)							
Sizzle + Selective	21	26.3	4.8	35.2	8.3	0.001 *	0.001 *
Health Promotion in Nursing	36	28.3	10.0	42.1	6.2	0.001 *	0.001 *
PHABB	17	31.7	5.9	46.1	4.5	0.001 *	0.001 *
KAP: Confidence in Counseling (11 questions)							
Sizzle + Selective	20	29.4	6.2	39.0	8.0	0.001 *	0.004 *
Health Promotion in Nursing	37	31.2	8.3	44.3	6.3	0.001 *	0.001 *
PHABB	17	32.4	5.1	47.0	5.2	0.001 *	0.001 *
KAP: Knowledge about Obesity ^c^ (17 questions)							
Selective ^d^	12	13.6	2.2	13.5	1.8	0.89	0.59
Health Promotion in Nursing	33	10.9	2.2	12.1	2.2	0.01 *	0.01 *
PHABB	17	12.4	1.5	14.0	1.6	0.001 *	0.002 *

^a^ KAP = Knowledge, Attitudes, Practice. Scoring: 1 (strongly disagree) to 5 (strongly agree). ^b^ Due to small numbers, Sizzle and Selective were combined. ^c^ Scoring: number of questions answered correctly. ^d^ Could not include Sizzle due to difference in number of questions. ^e^ *p*-value computed from paired *t*-tests for continuous variables using matched pairs. ^f^ *p*-value computed from Wilcoxon signed-rank test of difference between pre and post measures. * *p* < 0.05.

**Table 3:** Pre-course and post-course survey measuring health professional trainees’ self-reported lifestyle behaviors (categorical variables).

		Nursing					PHABB					Selective				
		Pre		Post		*p*-Value ^a^	Pre		Post		*p*-Value	Pre		Post		*p*-Value
Variable	Category	n	%	n	%		n	%	n	%		n	%	n	%	
Days eat	0–1 days	15	42.9	4	11.0		2	10.5	3	16.0		7	43.8	1	6.0	
5 servings	2–3 days	11	31.4	20	57.0		9	47.4	3	16.0		5	31.3	8	50.0	
fruits and	4–5 days	7	20.0	6	17.0		5	26.3	11	58.0		2	12.5	6	38.0	
veggies	6–7 days	2	5.7	5	14.0	0.007 *	3	15.8	2	11.0	0.44	2	12.5	1	6.0	0.01 *
Consume	0 < 25%	14	40.0	10	29.0		3	15.8	1	5.0		5	31.3	1	6.0	
whole	25–<50%	6	17.1	5	14.0		6	31.6	7	37.0		4	25.0	5	31.0	
grains	50–<75%	8	22.9	13	37.0		7	36.8	6	32.0		5	31.3	8	50.0	
	75–100%	7	20.0	7	20.0	0.16	3	15.8	5	26.0	0.15	2	12.5	2	12.0	0.03 *
Advise	No	29	82.9	23	66.0		1	5.3	2	11.0		14	87.5	15	94.0	
patients	Yes	1	2.9	8	23.0		14	73.7	17	89.0		1	6.3	1	6.0	
about	Maybe	1	2.9	2	6.0		4	21.1	0	0.0		1	6.3	0	0.0	
nutrition	Don’t Know	4	11.4	2	6.0	0.75	0	0.0	0	0.0	0.09	0	0.0	0	0.0	0.98
Advise	No	30	85.7	25	71.0		12	63.2	11	58.0		15	93.8	16	100.0	
patients	Yes	1	2.9	7	20.0		5	26.3	3	16.0		0	0.0	0	0.0	
about	Maybe	1	2.9	1	3.0		2	10.5	5	26.0		0	0.0	0	0.0	
cooking	Don’t Know	3	8.6	2	6.0	0.63	0	0.0	0	0.0	0.27	1	6.3	0	0.0	0.98
Mins/day	<10 min	0	0.0	1	3.0		2	10.5	1	5.0		1	6.3	1	6.0	
moderate	20 min.	5	14.3	8	23.0		4	21.1	5	26.0		2	12.5	6	38.0	
exercise	30 min.	9	25.7	9	26.0		5	26.3	3	16.0		5	31.3	2	12.0	
	40 min	4	11.4	5	14.0		2	10.5	3	16.0		4	25.0	2	12.0	
	50 min	1	2.9	4	11.0		2	10.5	2	11.0		0	0.0	1	6.0	
	60 min	13	37.1	8	23.0		3	15.8	5	26.0		4	25.0	4	25.0	
	90 min	3	8.6	0	0.0		0	0.0	0	0.0		0	0.0	0	0.0	
	120 min	0	0.0	0	0.0		0	0.0	0	0.0		0	0.0	0	0.0	
	150 min +	0	0.0	0	0.0	0.06	1	5.3	0	0.0	0.71	0	0.0	0	0.0	0.60
Hours	<5 h	0	0.0	7	20.0		0	0.0	0	0.0		1	6.3	0	0.0	
per day	5–6 h	13	37.1	9	26.0		2	10.5	0	0.0		2	12.5	3	19.0	
sleeping	6–7 h	13	37.1	13	37.0		10	52.6	5	26.0		5	31.3	4	25.0	
	7–8 h	9	25.7	5	14.0		5	26.3	10	53.0		6	37.5	7	44.0	
	8+ h	0	0.0	1	3.0	0.05	2	10.5	4	21.0	0.01 *	2	12.5	2	12.0	0.42

^a^ *p*-value derived from χ^2^ test for categorical data. * *p* < 0.05.

**Table 4:** Pre-course and post-course survey measuring health professional trainees’ self-reported lifestyle behaviors (continuous variables).

			Pre-Class		Post-Class					
Variable	Class Group	n	Mean	Std Dev	Mean	Std Dev	t	*p*-Value ^a^	Wilcox	*p*-Value ^b^
Days/week	Health Promotion in Nursing	35	4.1	1.6	4.0	1.6	0.5	0.62	124.5	0.68
moderate	PHABB	19	2.9	1.6	4.0	1.4	4.2	0.001 *	0.0	0.003 *
exercise	Selective	16	3.4	2.0	3.3	1.6	0.3	0.79	42.0	0.49
Work-Life	Health Promotion in Nursing	35	3.0	0.8	3.1	0.9	0.5	0.64	76.0	0.64
Balance	PHABB	19	2.8	0.9	3.7	0.8	4.3	0.001 *	5.0	0.002 *
Scale	Selective	16	3.3	0.8	3.3	1.2	0.0	0.98	18.0	0.98
Social Well-	Health Promotion in Nursing	35	3.5	0.9	3.5	1.0	0.2	0.86	132.0	0.98
Being	PHABB	19	3.2	0.7	3.9	0.8	3.4	0.003 *	4.0	0.008 *
Scale	Selective	16	3.6	1.0	3.8	1.1	1.0	0.33	15.0	0.32
Days/week	Health Promotion in Nursing	35	4.9	2.0	5.1	1.5	0.8	0.43	94.0	0.45
Cook	PHABB	19	3.6	1.9	4.0	1.7	1.1	0.27	29.5	0.25
Dinner	Selective	16	4.7	1.5	5.0	1.8	0.8	0.41	19.0	0.38
Days eat	Health Promotion in Nursing	35	2.3	1.8	2.0	1.6	1.1	0.26	104.5	0.29
take-out	PHABB	19	3.4	1.9	2.8	1.6	1.5	0.15	34.0	0.13
	Selective	16	2.8	2.0	2.5	1.8	0.8	0.45	23.0	0.36
Days eat	Health Promotion in Nursing	35	3.5	2.4	3.1	2.2	1.1	0.29	105.0	0.19
home cook	PHABB	19	3.0	2.0	3.2	1.8	0.5	0.59	32.5	0.35
dinner	Selective	16	4.1	2.1	4.4	1.8	0.7	0.48	36.0	0.50
Hours/wk	Health Promotion in Nursing	35	6.2	0.9	5.9	1.0	2.3	0.03 *	93.5	0.03*
sleep	PHABB	19	6.8	0.7	6.8	0.8	0.1	0.89	43.5	0.89
weekdays	Selective	16	7.0	1.0	7.0	0.9	0.5	0.63	12.0	0.73
Hours/wk	Health Promotion in Nursing	35	7.7	1.1	7.8	1.4	0.8	0.42	181.0	0.42
sleep	PHABB	19	7.9	0.9	8.4	0.6	2.3	0.03 *	10.0	0.04*
weekend	Selective	16	7.4	1.2	7.5	0.6	0.1	0.90	28.5	0.69
Hours/wk	Health Promotion in Nursing	35	1.1	1.5	0.7	0.8	1.5	0.13	50.5	0.21
napping	PHABB	19	0.2	0.5	0.2	0.5	0.2	0.85	5.0	0.98
	Selective	16	0.1	0.3	0.1	0.3	1.8	0.05	8.0	0.05
Epworth	Health Promotion in Nursing	35	9.4	4.2	8.2	4.8	1.9	0.06	129.5	0.09
Sleepiness	PHABB	19	6.3	4.0	5.0	3.2	2.3	0.03 *	29.0	0.04 *
Scale	Selective	16	0.1	0.3	1.0	0.3	1.5	0.16	0.0	0.16

^a^ *p*-value computed from paired *t*-tests for continuous variables using matched pairs. ^b^ *p*-value computed from Wilcoxon signed-rank test of difference between pre and post measures. * *p* < 0.05.

**Table 5:** Pre-course and post-course survey measuring health profession trainees’ culinary skills and nutrition literacy.

		Pre-Class		Post-Class					
Outcome Measure Class Group	n	Mean	Std Dev	Mean	Std Dev	*t*-Test	*p*-Value ^a^	Wilcoxon	*p*-Value ^b^
Cooking Skills Questions (max = 60)									
Health Promotion in Nursing	31	31.84	10.11	41.26	8.41	−7.72	0.0000 *	−219.50	0.0001 *
PHABB	8	38.25	8.84	45.38	6.32	−4.06	0.0048 *	−17.00	0.0156 *
Selective	11	34.27	7.88	48.00	7.52	−7.22	0.0001 *	−33.00	0.0010 *
Nutrition Literacy Questions (max = 30)									
Health Promotion in Nursing	31	15.52	4.93	20.61	4.32	−7.99	0.0001 *	−175.5	0.0001 *
PHABB	8	18.13	2.80	23.00	1.51	−5.45	0.0010 *	−18.00	0.0078 *
Selective	11	15.36	3.83	24.36	3.44	−8.48	0.0001 *	−33.00	0.0010 *

^a^ *p*-value computed from paired *t*-tests for continuous variables using matched pairs. ^b^ *p*-value computed from Wilcoxon signed-rank test of difference between pre and post measures. * *p* < 0.05.

## Data Availability

Research data can be requested from the authors.

## Supplementary Materials

The following supporting information can be downloaded at: https://www.mdpi.com/article/10.3390/nu15194240/s1, Table S1: Survey about “Provider’s Role in Managing Obesity,” “Confidence in Obesity Knowledge,” and “Confidence in Counseling Families about Obesity”.
