# Supplementary material for: Impact of a Teaching Kitchen Curriculum for Health Professional Trainees in Nutrition Knowledge, Confidence, and Skills to Advance Obesity Prevention and Management in Clinical Practice
Source: Nutrients. 2023 Sep 30;15(19):4240. doi: 10.3390/nu15194240 (PMC10574719; doi:10.3390/nu15194240)
Supplement: Supplementary file 1 [file nutrients-15-04240-s001.zip › nutrients-2600223-supplementary.pdf]

Table S1: Survey about “Provider’s Role in Managing Obesity,” “Confidence in Obesity Knowledge,” and “Confidence in Counseling Families about Obesity”

“Provider’s Role in Managing Obesity”

How much do you agree or disagree with the following statements:	Strongly Agree	Agree	Neither Agree nor Disagree	Disagree	Strongly Disagree
A. It is the primary care provider’s role to identify obesity in children.	5	4	3	2	1
B. It is the primary care provider’s role to provide dietary counseling to children and families.	5	4	3	2	1
C. Primary care providers can be effective in treating childhood obesity.	5	4	3	2	1
D. It is the primary care provider’s role to counsel on healthy cooking strategies to children and families.	5	4	3	2	1
E. It is the primary care provider’s role to support and counsel on breastfeeding.	5	4	3	2	1
F. It is the primary care provider’s role to identify community resources that exist for children who are overweight or obese.	5	4	3	2	1
G. It is the primary care provider’s role to counsel families on the benefits of cooking as it relates to emotional, physical and social well-being.	5	4	3	2	1
H. It is the primary care provider’s role to collaborate with WIC centers to promote healthy eating strategies for young children.	5	4	3	2	1
I. It is the primary care provider’s role to identify and learn the federal, state, and local food policies that influence eating for children and families.	5	4	3	2	1

“Confidence in Obesity Knowledge”

I feel confident in my knowledge to:	Strongly Agree	Agree	Neither Agree nor Disagree	Disagree	Strongly Disagree
A. Describe the prevalence of childhood obesity and overweight by age group.	5	4	3	2	1
B. Describe the prevalence of childhood obesity by race/ethnic group.	5	4	3	2	1

C. Discuss with patients the American Academy of Pediatrics' recommendations for breastfeeding mothers.	5	4	3	2	1
D. Discuss with patients evidence-based nutrition related recommendations to prevent childhood overweight and obesity.	5	4	3	2	1
E. Discuss with patients evidence based dietary guidelines for specific weight related comorbidities.	5	4	3	2	1
F. Identify age-specific promoters and detractors of healthy eating in childcare, school, and university settings.	5	4	3	2	1
G. Define food literacy and discuss the determinants of food literacy.	5	4	3	2	1
H. Discuss federal, state and local nutrition/food policies that influence eating for children and families.	5	4	3	2	1
I. Discuss how implicit weight bias can influence your own and others physical, emotional and social wellbeing.	5	4	3	2	1
J. Define food insecurity and discuss the determinants of food insecurity.	5	4	3	2	1
K. Identify the association between decreased sleep, physical activity, and other daily routines and obesity risk in children and young adults.	5	4	3	2	1

"Confidence in Counseling Families about Obesity"

**I feel confident in my abilities to:**	**Strongly Agree**	**Agree**	**Neither Agree nor Disagree**	**Disagree**	**Strongly Disagree**
A. Make a difference in my patients' diet and eating habits.	5	4	3	2	1
B. Counsel my patients using open- ended questions.	5	4	3	2	1
C. Counsel families on age-specific parenting skills to help support target behaviors around patient diet/eating and physical activity.	5	4	3	2	1

D. Know what community resources exist for children who are overweight or obese.	5	4	3	2	1
E. Counsel families using evidence based dietary guidelines for specific weight related comorbidities.	5	4	3	2	1
F. Use motivational interviewing to influence eating habits and behaviors.	5	4	3	2	1
G. Counsel on how to use cooking strategies to engage the whole family in behavior changes related to nutrition.	5	4	3	2	1
H. Counsel on the benefits of cooking as it relates to family food literacy.	5	4	3	2	1
I. Counsel families on age-specific hunger and satiety regulation.	5	4	3	2	1
J. Counsel on the benefits of cooking as it relates to emotional, physical and social well-being.	5	4	3	2	1
K. Screen and assess common mental health conditions associated with childhood obesity.	5	4	3	2	1
